# General Hospitals

**Published:** 1892-08-27

**Authors:** 


					Aug. 27, 1892. THE HOSPITAL. 365"-
The Institutional Workshop.
HOSPITAL ADMINISTRATION.
I.-
-GENERAL HOSPITALS.
?c or many years past those most interested in tne ad-
ministration of our voluntary hospitals have desired to
8ecu*e the general adoption of some plan which would
make it possible to compare results upon an identical
basis. No one interested in the attainment of sound
administration can fail to be impressed by the difficul-
ties of making thorough comparisons between the
relative success of various systems in institutions of the
same size and importance. There is no absence of
material, but each batch of facts has been compiled
ai*d put together upon a plan favoured by the compiler.
Sence it has been impossible to form a definite and
convincing judgment as to how it has come to pass that
0rie hospital expends half as much again, or twice as
much per bed, as another. In our issue of the 23rd
^nly, attention was called to one of the necessary
results of the voluntary system, which engenders a
sPecies of rivalry among the authorities as to which can
Prove itself most worthy of support. Unfortunately
o^ing to the absence of reliable data compiled upon an
^entical basis, the public often forms a wrong judg-
ment, and so the prize of liberal offerings sometimes
fells to the lot of those institutions which are not
among the best administered, although they may be
the most successful in enlisting public sympathy by
causing their work to be universally known.
One of the things most desired is the adoption of a
uniform system of accounts. The Committee of Dis-
tribution of the Hospital Sunday Fund have conferred
^ith the secretaries of the leading metropolitan hos-
pitals, and so a general agreement has been arrived at,
and a uniform system of accounts has been formulated
and approved. A few months ago the Council of the
Metropolitan Hospital Sunday Fund agreed to recom-
mend this uniform system for general adoption. In
the result very many hospitals have agreed to keep their
books uDon an identical system, and so more uniformity
has been secured. Unfortunately, certain of the hos-
pitals, either because they do not understand how easy
it is to modify a system of accounts so as to bring them
into line with the uniform system referred to, or
because they are too lazy or too indifferent to pubuj
?pinion to at once avail themselves of the undoubted
advantages which would arise from its adoption, have
Bo far failed to accept the recommendation of the Com-
mittee of the Hospital Sunday Fund.
At the meeting of the Council of the Sunday Fund
at the Mansion House on Friday, the 29th ultimo, atten-
tion was called to the fact that certain of the hospitals
bad not yet adopted this uniform system. Although
110 action was then taken, because, as Sir Sydney
^aterlow expressed it, the Council hoped that moral
suasion and patience would induce the sluggards to
come into line, yet it was urged, and urged with much
force by more than one speaker, that ultimately the
Question must arise as to whether any institution which
failed to present its accounts in the form required by the
hospital Sunday Fundshould in future receive any grant
from the fund. The Hospital Saturday authorities
bave also, we understand, determined to adopt the same
uniform system of accounts, and we would earnestly
urge the committees and officers of any hospital which
has not yet agreed to make the necessary modifications
in their accounts to take this step promptly in the best
interests of their institution, and for the sake of their
own credit and security.
The Expenses of Management.
The Metropolitan Hospital Sunday Fond has for
some years past prepared tables showing the number of
beds occup ed daily each year; the number of home
visits from dispensaries; the number of cases treated,
with approximate cost of treatment; the percentage
of the cost of administration as compared with that of
maintenance; the average amount of patients' pay-
ments ; and the average gross annual income and ex-
penditure during three years.
We propose to examine the percentage of the cost of
administration as compared with that of maintenance,-
as shown in these returns. Taking the large general
hospitals first, we find the facts to be as follows :?
Name of Hospital.
London ...
Gay's
St. George's
Middlesex
St. Mary's
Seamen's
King's
University
Westminster
Charing Croes
Royal Free
German
Training (Totten-
ham)
West London
London Homoeo-
pathic
Percentage of
cost of
Administra-
tion to that of
Main enarce.
8,842
6,136
4,054
3,018
3 923
3,152
2,075
3 465
2,557
2,291
1,866
1,301
798
6 047
7-600
4-892
7 162
6 667
11-663
12-551
14 829'
6-782
12241
7 240
13-235
5-982
11 877
18-716
The foregoing table contains the names of all the
general hospitals which have 100 beds and upwards.
We have added the " London Homoeopathic " to the
list because it has a medical school, and is usually in-
cluded, although it only contains 94 beds. Besides,
the Governors have determined to build a new hospital,
which will contain more beds than are at present
available.
The management of the voluntary hospitals has
often been attacked on the ground of its extravagance.
Over and over again the Editor of the Hospital
Annual has been asked by the lay Press to show the
cost of management per cent., as set forth in the above
table. The difficulties in the way of preparing a fair
statement on this head are very great, but the above
figures may be taken as impartial, and accurate, seeing
that they have been determined by the Committee of
Distribution of the Hospital Sunday Fund.
A careful study of the circumstances of the various
hospitals, and of the expenditure which it is essential
that each committee shall sanction in order to secure
the maximum of efficiency in administration, leads us to
the conclusion, that 15 per cent, represeats on the
whole the maximum amount which the cost of ad-
-r-
366 THE HOSPITAL, Aug. 27, 1892.
ministration ought to bear to that of maintenance in
any general hospital containing 100 beds and upwards.
Judged by this standard it will be seen that none
of the general hospitals in London exceed this
limit, whilst very many of them expend far less. It
is true that the percentage of the cost of admi-
nistration to that of maintenance at the London
Homoeopathic is 18'716 per cent. It must be re-
membered, however, that this is a relatively small
institution, the only representative of its class,
and that possibly under these circumstances, the
struggle to live is far more severe than in the case of
the larger and older general hospitals. In any case it
will be interesting to hear what explanation the authori-
ties have to give of the causes which have necessitated
a relatively larger expenditure upon administration in
the case of this institution than has been found neces-
sary by the committees of the other hospitals included
in the table.
We do not think that tbe Training Hospital, Totten-
ham, can be fairly compared with the other institutions,
seeing that it is managed?we believe?by a sisterhood,
and that much of the service is rendered either freely
or for a nominal payment. We congratulate the authori-
ties of St. George's Hospital on the fact that they ex-
pend less than 5 per cent, upon management, although
we are of opinion that they could, with advan-
tage, spend a considerably larger sum than they do at
present upon salaries, an item which, rightly under-
stood, produces results so far as efficiency in the admin-
istration is concerned, in direct proportion to the per-
centage which it bears to a liberal and wise expenditure.
The London expends barely 6 per cent., while it will
he seen that there is almost a tie between St. Mary's
and the Westminster Hospitals, both of which expend
less than 7 per cent, on management. There are
no better managed hospitals in London than the
London, Westminster, and St. Mary's, a fact which
makes the smallness of this expenditure not only
creditable, but remarkable. The Middlesex and the
Uoyal Free expend rather more than 7 per cent.,
and Guy's rather more than per cent,
upon management. Next in order follow the
Seamen's and the West London, which each expend
more than 11? per cent.; Charing Cross, which expends
12? per cent.; King's College, 12J per cent.; the Ger-
man, 13? per cent.; and University College, 14-829 per
cent, respectively.

				

